# Integrative analysis of multiple genomic data from intrahepatic cholangiocarcinoma organoids enables tumor subtyping

**DOI:** 10.1038/s41467-023-35896-4

**Published:** 2023-01-16

**Authors:** Hee Seung Lee, Dai Hoon Han, Kyungjoo Cho, Soo Been Park, Chanyang Kim, Galam Leem, Dawoon E. Jung, Soon Sung Kwon, Chul Hoon Kim, Jung Hyun Jo, Hye Won Lee, Si Young Song, Jun Yong Park

**Affiliations:** 1 Division of Gastroenterology, Department of Internal Medicine, Yonsei University College of Medicine, Seoul, Korea; 2 Institute of Gastroenterology, Yonsei University College of Medicine, Seoul, Korea; 3 Division of Hepatobiliary and Pancreatic Surgery, Department of Surgery, Yonsei University College of Medicine, Seoul, Korea; 4 Department of Pharmacology, Yonsei University College of Medicine, Seoul, Republic of Korea

**Keywords:** Bile duct cancer, Bile duct cancer, Cancer models

## Abstract

As genomic analysis technology has advanced, it has become possible to sub-classify intrahepatic cholangiocarcinoma (ICC) at the histological or molecular level. Here, we verify the recently suggested two subgroups of ICC in the organoids model, compare the characteristics between types. ICC patients are subclassified into small-duct (SD) and large-duct (LD) subtype according to histological characteristics. ICC organoids are established, and unsupervised principal component analysis clustering separates each type of ICC. Differential gene expression reveals enrichment on KRAS, TGFβ and ERBB2 signaling pathways in LD-type compared with SD-type (*P* < 0.05). Gene set enrichment analysis demonstrates that the cholangiocarcinoma class 2 signature, defined by Andersen et al., is enriched in the LD-type (enrichment Score = 2.19, *P* < 0.001). A protein-protein interaction network analysis identifies ZNF217 as a significant hub protein (odds ratio = 4.96, *P* = 0.0105). We perform prospective modeling of histological subtype using patient-derived organoids. Moreover, gene expression profiling of ICC organoids enables identification of type-specific targetable pathways.

## Abstract (web summary)

There is still need for representative models of intrahepatic cholangiocarcinoma (ICC) subtypes. Here, the authors develop organoids for two recently suggested ICC subtypes and identify distinct transcriptional profiles and potential therapeutic targets.

## Introduction

The incidence of cholangiocarcinoma, a group of bile duct cancers with a very poor prognosis, is increasing worldwide^1^. Intrahepatic cholangiocarcinoma (ICC), which has a 5-year survival rate of <20%, has shown a particularly rapid increase. The majority of such patients have already reached advanced stages at the time of diagnosis and are treated with palliative therapy^2,3^.

As genomic analysis technology has advanced, a grouping of ICCs at histological or molecular levels has become possible^4,5^. In particular, recent studies, and the 5th edition of the World Health Organization Classification of Tumors, classify ICCs into two types—large duct (LD) and small duct (SD)—based on histology, location, and key mutations^6–14^. Although tumor origin, carcinogenesis, and response to chemotherapy are expected to differ between the two types, in-depth studies of these differences have proven experimentally problematic owing to the absence of applicable ex vivo subtype models and difficulties in acquiring sufficient tumor tissue for analysis. Therefore, a truly representative model of cholangiocarcinoma subtypes is needed for studying differences in development, carcinogenesis, and anticancer drug responsiveness between types.

Recently developed organoid culture techniques enable the formation of adult stem cell-derived cancer organoids from cholangiocarcinoma specimens^15–20^. Importantly, the resulting organoids show similar phenotypic and genetic characteristics compared with the original primary tumor. A previous study showed that even small biopsy samples are sufficient for the generation of tissue-derived organoids^21^. Despite these advances, no studies have yet applied ICC organoids to subtype analysis of ICC.

Here, we show an ex vivo model that reflects the molecular pathogenesis and genetic properties of the corresponding primary ICC. We further identify potential type-specific targetable pathways in ICC patients for future personalized therapy.

## Results

### ICC organoids exhibit characteristics of the patient’s primary tumor

Cancer tissues, matching normal tissues, and blood samples were collected from 16 patients diagnosed with cholangiocarcinoma, as confirmed by histological analysis. Tissues were acquired by surgical resection (*n* = 5) or biopsy (*n* = 11). Median tumor size was 5.2 cm (range, 2.0–15.0 cm). Of the 16 patients, 11 (68.8%) were male and 5 (31.2%) were female. Baseline characteristics are described in Table 1.

**Table 1:** Patients baseline characteristics

Organoid	CA 19-9 (U/mL)	Size (cm)	Distant metastasis	Stage	Acqusition method	Surgery/ chemotherapy name	Grade	PD-L1	Cirrhosis	Viral hepatitis	Cholelithiasis	Choledocholithiasis	PSC	Best response	Time to progression (days)	Time to recurrence (days)
YCO-1	24	3.8	None	pT1aN0M0	Surgery	Right lobectomy	G2	Not done	Yes	0	0	0	0			421
YCO-2	906	6.4	Peritoneum	cT1bN1M1	Biopsy	Best supportive care	G2	Not done	0	0	0	0	0			
YCO-3	381	6.2	Lung, adrenal	cT1bN1M1	Biopsy	Gemcitabine/cisplatin	G3	TPS 50%	0	HBV	0	Yes	0	PD	58	
YCO-4	53	4.5	Aortocaval lymph node	cT1aN1M1	Biopsy	Gemcitabine/cisplatin	G2	TPS 0%	0	0	0	0	0	PR	594	
YCO-5	39	14.4	None	cT2N0M0	Biopsy	Gemcitabine/cisplatin	G2	TPS 0%	0	0	0	0	0	SD	423	
YCO-6	1643	15.0	None	cT2N0M0	Biopsy	Gemcitabine/cisplatin	G2	TPS 1%	Yes	0	0	0	0	PD	60	
YCO-7	8	4.3	None	pT2N1M0	Surgery	Right lobectomy	G3	TPS 10%	0	HCV	0	0	0			102
YCO-8	17	9.5	None	pT1bN0M0	Biopsy	Right lobectomy	G3	Not done	0	0	0	0	0			499
YCO-9	251	5.2	Aortocaval lymph node	cT1bN1M1	Biopsy	Gemcitabine/cisplatin	G2	TPS 2%	Yes	HBV	0	0	0	PD	49	
YCO-10	94	5.7	Bone	cT1bN1M1	Biopsy	Gemcitabine/cisplatin	G3	TPS 60%	Yes	HBV	0	0	0	PD	51	
YCO-11	9345	5.0	None	cT2N1M0	Biopsy	5-Fluorouracil/cisplatin	G2	Not done	Yes	0	0	0	0	PD	63	
YCO-13	616	2.3	None	cT3N0M0	Biopsy	Best supportive care	G2	TPS 1%	0	0	0	0	0			
YCO-14	20,000	6.0	None	cT1bN1M0	Biopsy	Gemcitabine/cisplatin	G3	TPS 5%	0	0	0	0	0	SD	130	
YCO-15	4247	8.4	None	pT2N1M0	Surgery	Right hepatectomy	G2	TPS 1%	0	0	0	0	0			196
YCO-18	896	2.2	None	pT2N1M0	Surgery	Left lobectomy	G2	TPS 10%	0	0	0	0	0			87
YCO-19	247	2.0	None	pT2N1M0	Surgery	Segmental resection	G3	Not done	0	0	0	0	0			215

*TPS* tumor proportion score, *HBV* Hepatitis B virus, *HCV* Hepatitis C virus, *PD-L1* programmed death-ligand 1, *PSC* primary sclerosing cholangitis.

**Fig. 1:**
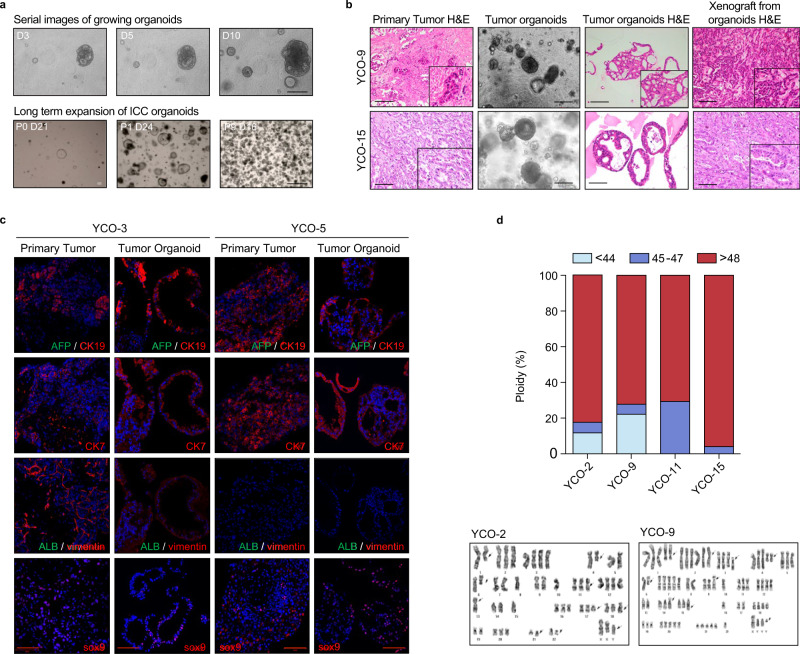
Establishment of ICC organoids and demonstration of their phenotypic similarity to corresponding primary tumors. **a** Time course of organoid growth (*n* = 16). Scale bar: 200 µm. **b** Representative matching images of H&E-stained primary tumors and organoids, showing the morphological and histological similarity of ICC organoids to their matching primary cancer (*n* = 16). Scale bar: 100 µm. **c** IF staining for the cholangiocyte ductal markers CK19 and Sox9 (red) and liver cell markers AFP and albumin (green), confirming preservation of primary tumor marker expression patterns in organoids (*n* = 2). Scale bar: 100 µm. **d** Representative image of normal tissue and tumor organoid karyotyping by GTG banding analysis, demonstrating aneuploidy in tumor organoids. ICC intrahepatic cholangiocarcinoma. Source data are provided as a Source Data file.

Organoids were established as described in the “Methods” section. Organoids usually appeared within 1 or 2 weeks after seeding. Thereafter, their size increased and they were passaged every 2 to 4 weeks (Fig. 1a). Organoids displayed various morphologies, such as cystic thin wall, cystic thick wall or compact type (Supplementary Fig. 1 and Supplementary Table 1), that were phenotypically similar to the primary tumor from which they were derived. The phenotypic similarity of organoid-derived xenografts to their corresponding primary tumor was also demonstrated by H&E staining (Fig. 1b and Supplementary Fig. 2) and immunofluorescence (IF) analysis, the latter of which demonstrated expression of the cholangiocyte ductal markers, KRT19 and SOX9, but not the liver cell markers AFP (alpha fetoprotein) and albumin (Fig. 1c) in both primary tumors and organoids. To determine if ICC organoids retained the expression of PD-L1, we next analyzed expression of PD-L1 in our panel of cholangiocarcinoma organoids. The expression of PD-L1 level in primary tumor tissue was matched with it of tumor organoids (Supplementary Fig. 3). Tumor organoids were also successfully implanted in the mouse subcutaneous area (data now shown). Karyotyping of morphologically suspicious organoids, performed to differentiate cancer organoids, revealed abnormal chromosomal numbers in cancer organoids (Fig. 1d and Supplementary Fig. 4). Among total samples collected of this study, 16 samples successfully established and used for this study (16/23, 69.5%). Compared to control normal organoids, cancer organoids grew continuously without senescence (Supplementary Figs. 5 and 6). Also, after thawing, we were still able to culture cancer organoids successfully (Supplementary Table 2). And we found no significant difference in the morphological changes and histology, growth rate, immunology (PD-L1 expression on organoids), and response to drug by the organoids between early and late passages (Supplementary Fig. 7). Considering the organoid establishment rate, patients with established organoids showed shorter “time to progression” (178.5 ± 210.3 days vs. 499.1 ± 298.1 days, *P* value = 0.054) or “recurrence-free survival” (253.3 ± 169.6 days vs. 316.8 ± 110.1 days, *P* value = 0.460) than those who failed to establish organoids. However, these differences were not statistically significant. Therefore, the organoids establishment rate showed the tendency to be associated with aggressive features in tumor (Supplementary Fig. 8).

### ICC organoids enable the sub-classification of cholangiocarcinoma tumors

**Fig. 2:**
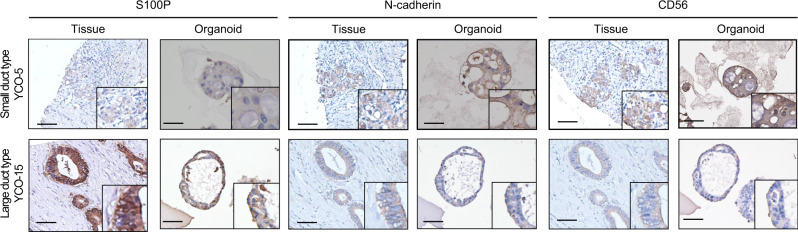
Characteristics of ICC organoids. ICC tumor subtype and paired organoid based on S100P, N-cadherin, and CD56 expression. LD type, S100P^+^; SD type, N-cadherin^+^CD56^+^ (*n* = 16). The LD type shows glanular and columnar cells in both tissue and organoids. The SD type exhibits compact, small round cells in priary tumor and organoids. (SD type, YCO-5; LD type, YCO-15). Scar bar, 100 μm.

It was recently shown that ICC can be subdivided into two groups based on S100P, N-cadherin and CD56 expression: LD type (S100P^+^) and SD type (N-cadherin^+^CD56^+^). Sub-classification is further aided by assessing the representation of glandular and columnar cancer cells. Histological analyses of primary tumors and matching organoids revealed compact, small, round cells in both tumors and matching organoids, and immunostaining confirmed the expected subtype classification of LD type (S100P^+^) and SD type (N-cadherin^+^CD56^+^) (Supplementary Table 1). This analysis showed that tumor tissue samples and organoid samples matched each other (Fig. 2b and Supplementary Fig. 9). To circumvent limitations posed by the tiny amounts of tumor tissue, we further discriminated cholangiocarcinoma types using organoids. A previous study reported that cirrhosis and hepatitis B virus (HBV) are the conditions most closely associated with ICC in Asian countries^22^. In the current study, we found that 27.7% (5/18) of patients had liver cirrhosis and 22% (4/18) had a hepatitis virus, three with HBV and one with hepatitis C virus (HCV). Liver cirrhosis and viral hepatitis were more prevalent in patients with SD-type ICC than those with LD-type ICC (Table 1).

**Fig. 3:**
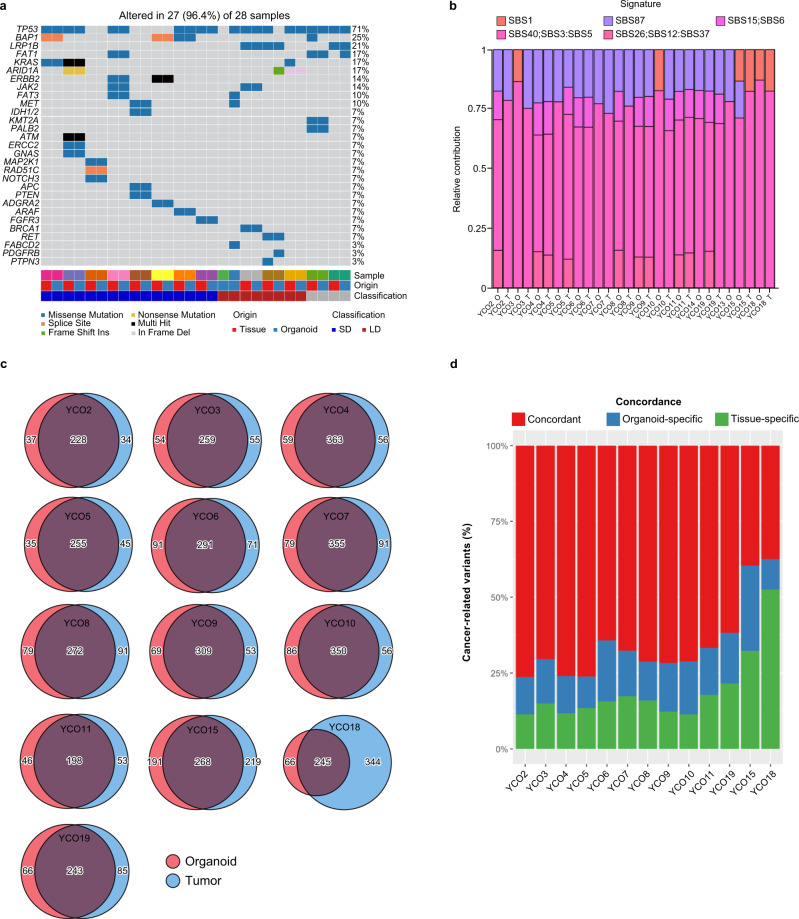
Whole exome sequencing. **a** Heatmap of somatic mutations in cholangiocarcinoma (*n* = 15). **b** Gene signatures according to each organoid and tumor (*n* = 15). **c** Somatic mutations shared between primary tumors and organoids. Venn diagrams show tumor organoid and primary tumor single nucleotide variants (*n* = 13). **d** The concordance (%) between the cancer-related variants found in the tumor of origin and the corresponding tumor organoids is shown by bar plots. Tissue-specific or organoid-specific mean that the cancer-related variants are discordant and not shared (*n* = 13).

We next evaluated the genetic similarities between original tumor specimens and matching organoids. We found that 27 of 28 samples (13 original tumor and 15 tumor organoids) (96.4%) exhibited somatic mutations, including mutations in tumor protein 53 (*TP53*; 71%), BRCA1-associated protein 1 (*BAP1*; 25%), the proto-oncogene GTPase *KRAS* (17%), AT-rich interaction domain 1A (*ARID1A*; 17%), and isocitrate dehydrogenase (*IDH*)-*1/2* (7%) (Fig. 3a). No fibroblast growth factor receptor 2 (*FGFR2*) translocations were identified in patients in this study. A high proportion of SD-type samples exhibited *BAP1* (37.5%) and *IDH1/2* (12.5%) mutations compared with LD type (0% for both) and single base substitution signature 1 (SBS1) was more frequently expressed in LD-type samples (Fig. 3b). Overall, this analysis showed that somatic mutations were shared between primary tumor specimens and organoids (Fig. 3c). An evaluation of the concordance of primary tumor mutations in tumor organoids with those in matching primary tumor specimens after germline variant removal showed that almost all samples showed concordance rates > 70% (Fig. 3d).

### Transcriptomic profiling identifies two distinct ICC subclasses according to duct type

**Fig. 4:**
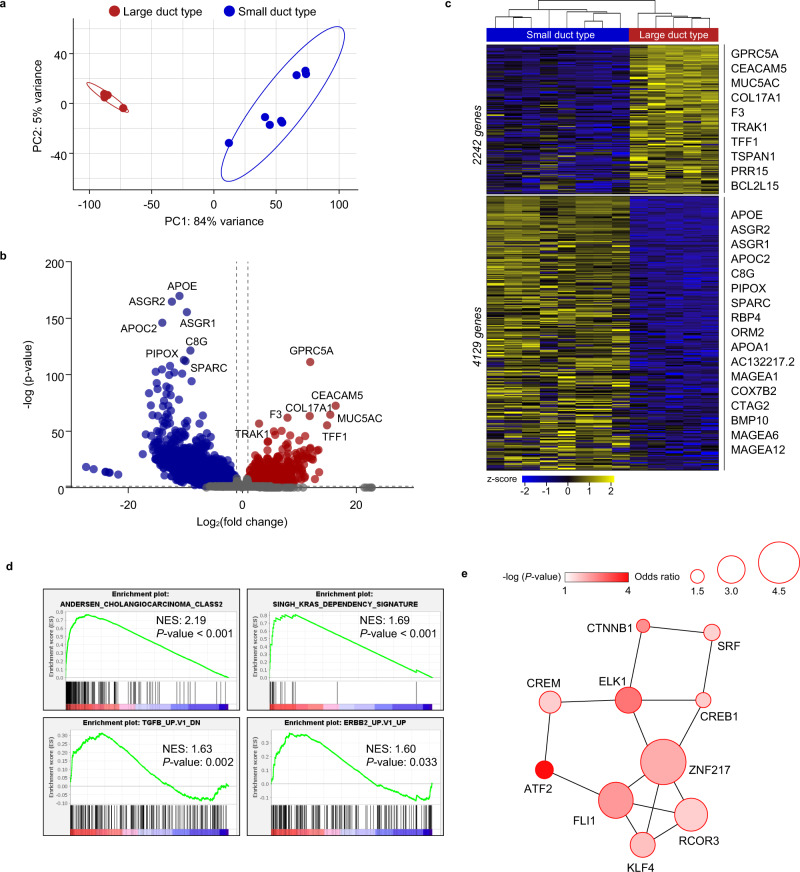
Transcriptome analysis of LD- and SD-type cancer organoids. **a** PCA plot showing clusters of samples according to transcriptional similarities (*n* = 13). **b**, **c** Volcano plot (**b**) and heatmap (**c**) showing differentially expressed genes (DEGs) between LD (red) and SD (blue) cancer organoids. DEGs were statistically calculated by DESeq method^51^ and defined by two criteria: the absolute value of log_2_(fold change) is >1 and the *P*-value is <0.05. A total of 2242 genes were up-regulated and 4129 genes were down-regulated in LD-type cancer organoids (*n* = 13). Representative genes are annotated. **d** GSEA of 2242 up-regulated genes in LD cancer organoids using gene sets released by the Molecular Signature Database (MSigDB). Four representative enrichment plots are shown: ‘cholangiocarcinoma class 2’, ‘KRAS dependency’, ‘TGFβ-up gene’, and ‘ERBB-up gene’ signatures. Normalized enrichment scores (NES) and *P*-values are indicated. Kolmogorov–Smirnov test. NES 2.19 and *P*-value <0.001 for the ‘cholangiocarcinoma class 2’ signature; NES 1.69 and *P*-value <0.001 for the ‘KRAS dependency’ signature; NES 1.63 and *P*-value 0.002 for ‘TGFβ-up gene’ signature; and NES 1.60 and *P*-value 0.033 for ‘ERBB-up gene’ signatures. **e** PPI network analysis of the top 10 transcription factors related to the 2242 up-regulated genes in LD-type organoids. We performed gene set enrichment analysis using the web-based software Enrichr^49,50^. The size of the circle represents the odds ratio, and the fill color represents the value of −log (*P*-value). The odds ratio and *p*-value for each key transcription factor are the following: ATF2 (activating transcription factor 2) odds ratio = 1.93, *P* < 0.0001; ELK1 (ETS transcription factor ELK1) odds ratio = 2.87, *P* = 0.0023; CTNNB1 (catenin beta 1) odds ratio = 1.47, *P* = 0.0046; FLI1 (friend leukemia integration 1 transcription factor) odds ratio = 3.97, *P* = 0.0062; ZNF217 (zinc finger protein 217) odds ratio = 4.96, *P* = 0.0105; KLF4 (Kruppel-like factor 4) odds ratio = 2.76, *P* = 0.0183; CREB1 (CAMP Responsive Element Binding Protein 1) odds ratio = 1.65, *P* = 0.0248; CAMP responsive element modulator (CREM) odds ratio = 2.38, *P* = 0.0259; REST Corepressor 3 (RCOR3) odds ratio = 3.61, *P* = 0.0267; and serum response factor (SRF) odds ratio = 1.78, *P* = 0.0285. NES normalized enrichment score.

Using RNA sequencing, we investigated distinctive transcriptome features according to the duct type (SD-type, *n* = 5; LD-type, *n* = 8) of the ICC organoid. SD-type and LD-type ICC organoids were readily classified according to their mRNA expression patterns (Fig. 4a). A comparison of the transcriptomes of LD-type and SD-type ICC organoids showed that 6371 genes were differentially expressed, 2242 of which were up-regulated in LD-type organoids and 4129 of which were down-regulated (Fig. 4b, c). *GPRC5A* (G protein-coupled receptor class C group 5 member A), *MUC5AC* (mucin 5AC, oligomeric mucus/gel-forming), and *TFF1* (trefoil factor 1) were highly expressed in LD-type organoids, and *APOE* (apolipoprotein E), *SPARC* (secreted protein acidic and cysteine-rich), and *BMP10* (bone morphogenetic protein 10) were highly expressed in SD-type organoids (Fig. 4b, c).

### Functional enrichment analysis and protein–protein interaction (PPI) network construction

We next performed a gene set enrichment analysis (GSEA) and found that, compared with SD-type ICC organoids, LD-type organoids exhibited significant enrichment of ‘cholangiocarcinoma class 2’, ‘KRAS dependency, ‘TGFβ-up gene, and ‘ERBB-up gene’ signatures (Fig. 4d). A PPI network analysis revealed that key transcription factors associated with LD-type ICC organoids included ATF2 (activating transcription factor 2) (odds ratio = 1.93; *P* < 0.0001), ELK1 (ETS transcription factor ELK1) (odds ratio = 2.87; *P* = 0.0023), CTNNB1 (catenin beta 1) (odds ratio = 1.47; *P* = 0.0046), FLI1 (friend leukemia integration 1 transcription factor) (odds ratio = 3.97; *P* = 0.0062), and ZNF217 (zinc finger protein 217) (odds ratio = 4.96; *P* = 0.0105) (Fig. 4e).

### ICC organoids predict patient’s treatment responses

**Fig. 5:**
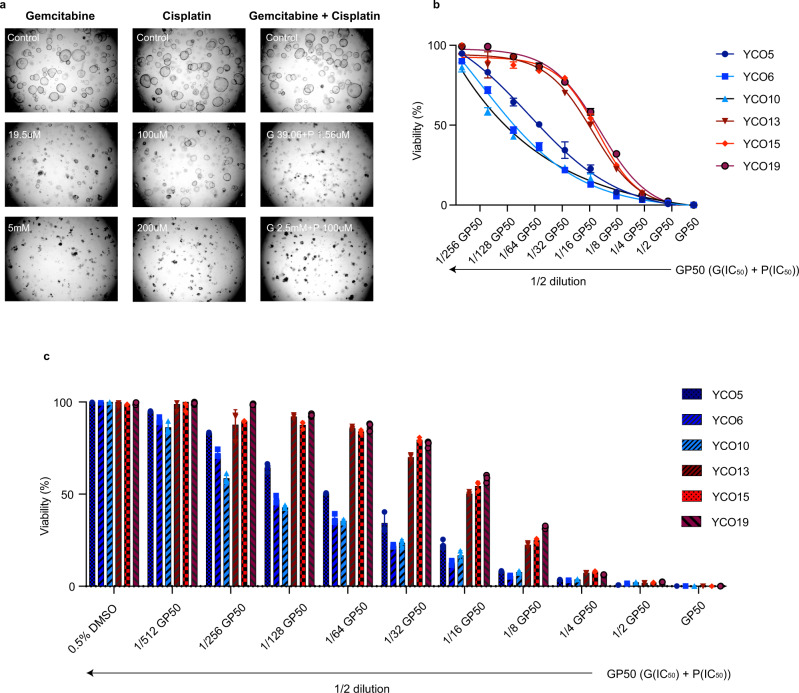
Chemotherapy responses of patients and patient-derived tumor organoids. Patient-derived organoids show duct-type–specific difference in cytotoxic drug sensitivity profiles. Scale bar: 200 µm. **a** Representative bright-field microscopy images of organoids (*n* = 6, 3 samples in LD- and SD-type, respectively). **b** Viability test showing differences in sensitivity to combined treatment with gemcitabine and cisplatin between LD (red) and SD (blue) cancer organoids. *n* = 6 biologically independent samples. Data are presented as mean ± SD (*n* = 6, 3 samples in LD- and SD-type, respectively). **c** Significant difference in IC_50_ values for the gemcitabine/cisplatin drug combination between LD- and SD-type organoids. *n*  =  6 biologically independent samples. Data are presented as mean ± SD. GP, gemcitabine and cisplatin (*n* = 6, 3 samples in LD- and SD-type, respectively). Source data are provided as a Source Data file.

In several previous studies, it has been shown that the SD type is associated with a better prognosis than the LD type^12,13^. Consistent with this, our drug response viability test revealed that IC_50_ values for the gemcitabine and cisplatin combination were higher for the LD type than for the SD type (*P* = 0.002) (Fig. 5a–c). An investigation of patient’s clinical data and drug responses of matching organoids showed a low IC_50_ value for gemcitabine/cisplatin (1.73 μM) in organoids from patient YCO-5, who was also a good responder (progression-free survival, 434 days), whereas the IC_50_ value for gemcitabine/cisplatin in organoids from patient YCO-15 was higher (11.16 μM), aligning with the fact that this patient was a poor responder (progression-free survival, 161 days). However, because the sample size for this study is small, simple comparisons by subtype between patients are of limited value. A further investigation of patients’ clinical data and tumor characteristics showed that SD-type patients had a larger median tumor size (6.9 cm) compared with LD-type patients (4.2 cm) and more advanced cancer stage regardless of their subtype.

## Discussion

In this study we established an ICC subtype model using an organoid system. We generated 16 ICC organoids that reflected the phenotype and genetic characteristics of the primary tumor. On the basis of the recent classification of ICC into subtypes, we subdivided all organoids into LD and SD types. We then confirmed that their type matched that of the primary tissue based on histologic appearance in H&E-stained samples. Using integrative clustering of multiple genomic data, we further identified several targetable gene pathways that distinguished LD and SD types. ICC organoids were already established in several previous studies; our study, however, was not conducted just to show the similarity of the organoids. Previous studies primarily depicted ICC organoids as a drug screening tool for the prediction of patient drug response and as a research tool for the discovery of biomarkers and therapeutic drugs (Supplementary Table 3). Fundamentally, we tried to find the differences in transcriptomic expression between large and small duct type ICC through the organoids model because the small number of tissues can limit the deep analysis and validation to discriminate subtypes. Three main points of this study were as follows: (1) it verified the recently suggested presence of two subgroups of ICC in the organoids model, (2) it compared the characteristics between LD and SD types of ICC, and (3) it found the type-specific gene expression profile and targetable pathway as a therapeutic target. Although ICC has been recently subdivided into LD and SD type, the developmental differences and treatment strategy remain to be explored. In addition, we investigated a larger number of patients compared to previous studies (16 patients vs. 3–4 patients), and we also used surgical resection and biopsy tissues.

Regarding ICC organoids, Broutier et al. reported that ICC organoids preserved the histological architecture, gene expression, and genomic landscape of the original tumor and suggested the possibility of subtype discrimination^15^. Nuciforo et al. showed that ICC organoids could be generated through needle biopsy technique and retained the morphology and tumor marker expression of the original tumors^21^. Recent several studies have reported that drug screening using patient-derived organoid identified potential therapeutic agents and prognostic biomarkers^18,20^. Up to now, however, there was no study that attempted to match the subtype of ICC with organoids. Notably, in-depth analyses have previously been largely intractable because only very small biopsy specimens are permitted for research purposes from most unresectable ICCs.

Recent standards divide ICC into two subtypes—SD and LD—based on their immunohistochemically evaluated phenotype. Patients with SD-type ICC usually have a history of chronic liver disease, and their tumors commonly harbor mutations in *IDH1, BAP1* and *FGFR2*, whereas patients with LD-type cancer have a history of premalignant biliary intraepithelial neoplasia, and their tumors most commonly show genetic changes in *SMAD4* and *KRAS*^7,9,11^. In the current study, eight patients could clearly be identified as SD type and five were identified as LD type. A few samples lacked sufficient primary cancer tissue to confirm subtype and their type was checked through the characterization of generated tumor organoids (YCO-5,14,15, and 19). Four samples were not used for further analysis due to difficult subtype classification. Consistent with a previous study, we confirmed that mutations in *TP53* and *BAP1* were more prominent in SD-type ICC. We further found that chronic liver disease was common among patients with SD-type ICC, and choledocholithiasis was common among LD-type patients. SD-type samples were primarily located in more peripheral liver locations compared with LD. Notably, histological and molecular findings of LD-type ICC in the present study are consistent with previously known characteristics of extrahepatic cholangiocarcinomas^23^, which are usually conventional adenocarcinomas with mucin production, diffuse S100P expression, and frequent *KRAS* mutations^10,24^.

Preclinical studies designed to translate molecular/cellular insights into therapeutic strategies are needed to overcome the poor prognosis in ICC. In this context, a number of studies have shown that not all biliary tract cancers are the same. Anderson et al. subdivided biliary tract cancer into classes 1 and 2 and showed a difference in prognosis between the two classes. An in-depth integrative analysis of tissues is required for such subtyping of biliary tract cancer, but tumors of many patients are unresectable, and the small amount of tissue acquired limits the ability to perform such analyses. By amplifying cancer epithelial cells through the generation of organoids, a small amount of initial tissue can ultimately yield a sample sufficient for integrative analysis and discovery of targetable pathways. In the current study, integrative clustering of multiple genomic data using organoid models showed that LD-type ICC corresponds to class 2 ICC in the Anderson classification scheme associated with poor prognosis. These results are consistent with previous studies showing that the prognosis of patients with LD-type ICC is poorer than that for patients with SD-type. LD-type ICC was also enriched for KRAS and TGFB signaling pathways, a finding similar to that reported by Jusakul et al. for extrahepatic cholangiocarcinoma^25,26^. Thus, the transcriptomic profile of the LD type is more similar to that of extrahepatic cholangiocarcinoma than is that of the SD type.

A recent Phase III clinical trial provides evidence supporting the use of IDH1 inhibitors and FGFR2 fusion inhibitors in cholangiocarcinoma (FIGHT-302, PROOF, and FOENIX-CCA3)^27–29^. Suggested targetable genes and anticancer drugs in cholangiocarcinoma have recently been released. Using our ex vivo model, promising drugs can be tested against morpho-molecular subclasses of organoids to predict drug efficacy before human clinical trials. In this study, patient YCO-8 harbored an *IDH* mutation, which can be targeted after disease progression; thus, this patient would be a candidate for ex vivo testing prior to clinical treatment.

With recent advances in genomic technology, targetable alterations are found in up to 34% of biliary tract cancers. In the current study, somatic mutations in *BAP1* were frequently detected in SD-type ICC. Recent studies have shown that *BAP1* acts as a tumor suppressor in ICC by modulating ERK1/2 and JNK/c-Jun pathways^30^. Clevers et al. demonstrated that loss of *BAP1* affects cell polarity and epithelial organization in human liver tissue and that *BAP1* is necessary for controlling chromatin accessibility of junctional and cytoskeletal genes. In an engineered human cancer organoid model, *BAP1* was shown to have an essential role in the development of malignant features^31^.

PPI network analyses demonstrated that ZNF217, a transcription factor suggested as a prognostic marker and therapeutic target in other types of cancer^32–35^, is a key hub protein in LD-type ICC. Notably, ZNF217 promotes epithelial–mesenchymal transition, invasion, and metastasis in cancer^32^. It can also attenuate apoptotic signals resulting from telomere dysfunction and may promote neoplastic transformation associated with chemotherapy resistance^36,37^. Accordingly, repressing ZNF217 expression inhibits cancer progression. Unfortunately, most studies on ZNF217 have been performed in ovarian and breast cancer, and none have yet been performed in cholangiocarcinoma. An integrative analysis of genomic data could establish ZNF217 as a prognostic and targetable marker in LD-type ICC, and its interactions could be involved in the poor prognosis of patients with LD-type ICC.

In this study, we aimed to develop a personalized, patient-derived, ICC-subtype–specific model. Previous studies have been limited with respect to type classification using surgical tissues, and the resulting lack of models has seriously constrained in-depth research on carcinogenesis and cancer development, and the discovery of targetable pathways. Thus, the organoid models developed in the current study can overcome limitations of tissue mass, simulate patient characteristics, and provide a platform for additional translational studies.

This study has several limitations. First, previous several studies have reported the establishment of ICC organoids or the classification of ICC and their molecular significance. Nonetheless, this study used large sample size and various sources including surgery and biopsy. Further, we investigated the type-specific gene expression profile and targetable pathway as a therapeutic target using organoids in order to overcome the limitation of molecular information by the small amount of tissues. Second, the frequencies of *IDH1/2*, *TP53* and *BAP1* mutations, which were more dominant in SD type than LD type, are higher than those in previous studies, which reported mutation frequencies of 32–41% for *TP53* and 4–6% for *IDH1/2*. These higher proportions may be explained by the increased variant allele frequency (VAF) of somatic mutations during organoid establishment. Previous studies have reported an increase in VAF of somatic mutation and an enhanced ability to detect it upon enrichment of cancer cells in patient-derived models^38,39^. Furthermore, it is likely that, given tumor heterogeneity and the small sample size used, the patients enrolled here do not represent full coverage of the ICC spectrum. Thus, future investigations employing larger sample sizes are warranted. Lastly, we were, unfortunately, unable to confirm the histological molecular subtype of all primary tumor tissues because of the low amount of primary tissue. Despite this, the present study was still able to investigate the correspondence between primary tumors and tumor-derived organoids and establish that tumor-derived organoids reflect the phenotype and characteristics of the primary tumor.

In conclusion, we performed prospective modeling of histological subtype specification in patient-derived ICC organoids. Through integrative clustering of multiple genomic data using this organoid model, we identified type-specific targetable pathways and characteristics.

## Methods

### Patients and samples

Cancer tissues matched normal tissues, and blood samples were collected from 16 patients who were pathologically diagnosed with cholangiocarcinoma based on biopsy or surgery (Table 1). All patients provided informed written consent, and procedures were approved by the Institutional Review Board of Severance Hospital according to ethical guidelines (IRB nos. 4-2018-0812 and 4-2018-1087). The collection and use of human samples were approved by the Ethics Committee of Severance Hospital, following the Declaration of Helsinki’s ethical guidelines. Tissues were transferred from the tissue-acquisition site to the laboratory within 2 h and placed in basal medium. If the amount of tissues was sufficient, tissues were divided for DNA and RNA sequencing and processing into formalin-fixed, paraffin-embedded blocks. All blood was separated into serum and plasma for DNA preparation for use in whole exome sequencing and further analysis. All tissues and blood were labeled and stored in a −70 °C freezer.

### Organoid culture

Cholangiocarcinoma organoids have been cultured with preserved histological architecture and genomic landscape of the original tumor^15,40,41^. We obtained tissue samples from surgery or biopsy, chopped and minced within 2 days after delivery, then washed and digested in digestion media from 1 h to overnight depending on the tissue acquisition method and condition. After digestion, tissues were washed with basal medium and seeded into Matrigel with organoid culture medium (composition described in Supplementary Table 4), which was changed every 3 days. We confirmed the established organoids as cancer based on four methods (H&E, Karyotype, Growth kinetics, and Somatic mutation) (Supplementary Figs. 2, 4, and 5).

### Immunohistochemistry and histological classification

Patients with cholangiocarcinoma were subdivided into SD- and LD-type according to their expression of the histological markers, S100P, N-cadherin and CD56, and cellular composition (Supplementary Table 5)^6–11^. SD type is composed of cuboidal to low columnar tumor cells arranged in acinar or small-sized tubular pattern and usually shows abundant N-cadherin and CD56 expression, but scant S100P expression. LD type presents as large-sized tubular or glandular components composed of tall columnar tumor cells and is usually characterized by abundant S100P expression and mucin production^6,8^. For immunohistochemistry, sections were counterstained with hematoxylin and imaged under a BX51 microscope (Olympus, Tokyo, Japan).

### Immunofluorescence

Cells were fixed in 4% paraformaldehyde (PFA) for 15 min at room temperature and washed three times with phosphate-buffered saline (PBS). Melted Histogel was then added to the organoid pellet and mixed carefully by pipetting, after which the HistoGel–organoid mixture was placed in a disposable base mold. The resulting HistoGel–organoid block was released from the mold, transferred into a paraffin block holder containing 10% neutral buffered formalin, incubated overnight at 4 °C, and then processed as a routine pathology specimen. For immunofluorescence analysis, paraffin-embedded sections were deparaffinized in xylene and rehydrated in a decreasing graded ethanol series. Antigen epitopes were then unmasked by heating samples in a 10 mM sodium citrate buffer (pH 6.0) according to standard procedures, after which sections were incubated overnight at 4 °C with primary antibodies. Primary antibodies against the following proteins were used: AFP (Abcam, ab3980, 1:200), KRT19 (Abcam, ab133496, 1:1000), KRT7 (Abcam, ab181598, 1:500), albumin (Bethyl Laboratories, A80-129A, 1:200), vimentin (Cell Signaling, 5741S, 1:400), SOX9 (Abcam, ab185966, 1:1000), and PD-L1 (Cell Signaling, 13684S, 1:1000). After incubation with primary antibodies, sections were incubated with species-appropriate Alexa Flour-conjugated secondary antibodies: Alexa Fluoro647 donkey anti-rabbit IgG (Invitrogen, cat. no. A31573, 1:2000), Alexa Fluoro488 donkey anti-mouse IgG (Invitrogen, cat. no. A21202, 1:2000), Alexa Fluoro594 donkey anti-goat IgG (Invitrogen, cat. no. A11058, 1:2000). Sections were lightly counterstained with 4’,6-diamidino-2-phenylindol (DAPI) and mounted. Fluorescent images were obtained using Zeiss LSM 780 (Zeiss, Germany).

### Karyotyping and chromosome counting

Cells were arrested in metaphase by adding 500 mL of colcemid (Gibco) stock solution and then incubating at 37 °C for 1 h in a humidified 5% CO_2_ atmosphere. Cells were subsequently collected into a 15-mL tube and then centrifuged at 738×*g*. for 10 min. The medium was carefully aspirated, and then 5 mL of hypotonic solution (0.075 M KCl) was added, and cells were allowed to stand at 37 °C for 25 min. Thereafter, 500 mL of Carnoy’s fixative (methanol:acetic acid = 3:1) was added, and the contents were mixed by inverting the tube. After the hypotonic solution was carefully aspirated, the supernatant was discarded, and the resulting pellets in approximately twice their original volume. were spread on prepared glass slides. Slides were baked at 60 °C for 30 min, treated with 50% H_2_O_2_ for 3 min, and then stained for G-bands using trypsin Giemsa stain (GTG banding).

### Whole exome sequencing

Genomic DNA was extracted from tumor tissues and organoids using an QIAamp DNA mini kit, according to the manufacturer’s instructions. After purification, DNA was eluted in 30 µL of water, and yield was determined using a Qubit DNA HS Assay (Thermo Fisher Scientific, Waltham, MA, USA), according to the manufacturer’s recommendations. The quality and quantity of purified DNA were assessed by fluorometry (Qubit; Invitrogen) and gel electrophoresis; a predefined yield of 300 ng of DNA was used as an acceptance criterion to ensure adequate library preparation. DNA-seq libraries were constructed using a TruSeq DNA exome Kit (Illumina Inc., San Diego, CA, USA) following the manufacturer’s recommendations. Briefly, 500 ng of genomic DNA from each sample was fragmented by acoustic shearing using a Covaris S2 instrument. Fragments (150–200 bp) were ligated to Illumina adapters and amplified by polymerase chain reaction (PCR). Samples were subsequently concentrated to 750 ng in 3.4 μL distilled water using a Speedvac (Thermo Scientific) and hybridized to RNA probes (SureSelect XT Canine All Exon V2 Kit Capture library; Agilent, Santa Clara, CA, USA) for 16–24 h at 65 °C. After hybridization, the captured targets were pulled down with biotinylated probe/target hybrids using streptavidin-coated magnetic beads (Dynabeads myOne Streptavidin T1; Life Technologies Ltd.) and appropriate buffers. The selected regions were then PCR-amplified using Illumina PCR primers. Libraries were identified with an Agilent TapeStation 4200 using High Sensitivity D 1000 ScreenTape (Agilent) and a KAPA Library Quantification Kit (Kapa Biosystems). High-quality libraries were pooled and sequenced (150 bp paired-end reads) on the NovaSeq6000 platform (Illumina) according to the manufacturer’s protocols. Image analysis was performed using NovaSeq6000 control Software version 1.3.1, and output base-calling data were de-multiplexed with bcl2fastq version v2.20.0.422, generating fastQC files.

Thereafter, sequencing reads were first mapped/aligned to reference genome hg19 using the Burrows–Wheeler Aligner (BWA). Pileup and variant calling from the aligned sequence reads were performed using BCFtools 1.12^42^, and called variants were annotated using ensembl variant effect predictor (VEP) release 105^43^. The resulting annotated variants were filtered according to the following criteria: depth (DP) > 50; alternate allele count (AC) > 5; and allele frequency (AF) > 0.05. The filtered variants and their effects on amino acid sequence were visualized using Maftools 2.10.0^44^.

### Transcriptomic analysis

Samples separated by duct type (SD-type and LD-type) were used for RNA sequencing. RNA sequencing was performed using a TruSeq Stranded mRNA Sample Prep Kit (Illumina). Adapter sequences and ends of reads with a Phred quality score <20 were trimmed, and reads shorter than 50 bp were simultaneously removed using cutadapt v.2.8^45^. Filtered reads were mapped to the species reference genome using the aligner, STAR v.2.7.1a^46^, following ENCODE standard options, with application of the “-quantMode TranscriptomeSAM” option for estimation of transcriptome expression level. Gene expression levels were estimated using RSEM v.1.3.1^47^ considering the direction of reads corresponding to the library protocol using the option “-strandedness”. Sequencing depth was normalized among samples by calculating FPKM (Fragments Per Kilobase of transcript per Million mapped reads) and transcripts per million (TPM) values. Differentially expressed genes (DEGs) were analyzed using the DESeq2 algorithm^48^ and were defined based on a *P*-value < 0.05 and log2 fold change > 1. Gene sets from the Molecular Signatures Database (MSigDB) were used for gene set enrichment analysis (GSEA), and protein–protein interactions for transcription factors were analyzed using the web-based software, Enrichr^49,50^.

### Establishment of xenografts from organoids

Exponentially growing organoids were trypsinized with TrypLE, then incubated in a 5% CO_2_ atmosphere at 37 °C, dispersed into single cells, and suspended in a 1:1 mixture of organoid culture medium and Matrigel (BD Biosciences). For evaluation of in vivo tumorigenicity, 2 × 10^6^ cells in 100 µcells in 100ty, 2rganoid culture medium and Matrigel (BD Biosciences). For evaluation of ment analysis (GSEA), and protein–protein interactions for transcription factors were analyzed. All mice were housed with 12 h dark/light cycle at room temperature (20–24 °C) with controlled humidity (40–60%). The maximal total volume for all tumors in mice, permitted by ethical protocol, was 2000 mm^3^, which was not exceeded. The mice were sacrificed by CO_2_ inhalation and tumors were harvested. Animals were housed at the Yonsei University animal care facility according to institutional guidelines. All experiments were performed in accordance with approved animal use procedures.

### Organoid histology

Organoids grown in Matrigel on coverslips were processed for histology and immunohistochemistry by first fixing in 4% PFA for 1.5 h, after which they were washed with PBS and stained with hematoxylin. Organoids were pre-embedded in Histogel (Richard Allen HG4000-012), returned to 10% neutral buffered formalin containing fixative, and incubated overnight. Organoids embedded in Histogel were processed with an automated tissue processor (Sakura VIP6) and embedded in paraffin blocks (Sakura Tissue-Tek TEC10). Samples were sectioned at 4 μm (Leica HistoCore BIOCUT) onto poly-l-lysine–coated slides and air-dried at 65 °C overnight for subsequent immunohistochemistry or routine hematoxylin and eosin (H&E) staining procedures. Histopathological features of ICC organoids were assessed using H&E staining and immunostaining for three markers: S100 calcium-binding protein P, N-cadherin, and neural cell adhesion molecule [NCAM]/CD56) (Supplementary Table 5). ICCs were classified into SD and LD types as described by previous studies^8,10^.

### Organoid drug sensitivity

Organoids were harvested from Matrigel and dissociated into single cells using TrypLE and mechanical dispersion. The cell suspension was resuspended in a 5% Matrigel/95% complete medium, after which 3000 cells were seeded per well of a 96-well plate and allowed to grow for 3 days. Organoid cultures were treated with different concentrations of gemcitabine and cisplatin, and the response of organoids to drugs was determined by measuring cell viability after 3 days of treatment using CellTiter-Glo 3D reagent (Promega) according to the manufacturer’s instructions. Data were analyzed and half-maximal inhibitory concentration (IC_50_) values were determined using GraphPad Prism (Version 9.1.0).

### Tumor response

Progression-free survival and responses of tumors to systemic chemotherapy were defined based on Response Evaluation Criteria in Solid Tumors (RECIST) v1.1 criteria using computed tomography (CT) or magnetic resonance imaging (MRI) findings.

### Statistical analysis

Continuous variables were expressed as means ± SDs, and categorical variables were expressed as proportions, *n* (%). Differences between groups with *P*-values < 0.05, determined by a Mann–Whitney *U* test, were considered statistically significant. For the data analysis and visualization, GraphPad Prism (Version 8.4.3), Adobe Illustrator CC 2022, and R (version 3.6.2) packages were used.

### Reporting summary

Further information on research design is available in the Nature Portfolio Reporting Summary linked to this article.

## Supplementary information

Supplementary Information

Reporting Summary

## Data Availability

The raw RNA-seq data generated in this study are available in the GEO database under accession code GSE215997. The raw whole exome sequencing data generated in this study are available in the GEO database under accession code GSE220940. Gene sets can be downloaded from MSigDB (https://www.gsea-msigdb.org/gsea/msigdb/). The remaining data are available within the Article, Supplementary Information, or Source data file. Source data are provided with this paper.

## Source data

Source Data
