# Supplementary material for: Integrative analysis of multiple genomic data from intrahepatic cholangiocarcinoma organoids enables tumor subtyping
Source: Nat Commun. 2023 Jan 16;14:237. doi: 10.1038/s41467-023-35896-4 (PMC9842736; doi:10.1038/s41467-023-35896-4)
Supplement: Supplementary file 1 — Supplementary Information [file 41467_2023_35896_MOESM1_ESM.pdf]

Supplementary Table 1. Organoids baseline characteristics

	Class	S100P	NCAD/CD56	Organoid Morphology
YCO-1	n/a	-	-	Compact
YCO-2	n/a	-	-	Compact and cystic
YCO-3	small duct type	0	1	Compact and cystic
YCO-4	small duct type	0	1	Compact
YCO-5	small duct type	0	1	Compact and cystic
YCO-6	indeterminate	-	-	Compact and cystic
YCO-7	small duct type	0	1	Compact
YCO-8	small duct type	0	1	Compact
YCO-9	small duct type	0	1	Compact
YCO-10	small duct type	0	1	Compact
YCO-11	small duct type	0	1	Compact
YCO-13	large duct type	1	0	Cystic
YCO-14	large duct type	1	0	Cystic
YCO-15	large duct type	1	0	Compact and cystic
YCO-18	large duct type	1	0	Cystic
YCO-19	large duct type	1	0	Cystic

Supplementary Table 2. Last passages of each sample and culture period

Sample name	Last passage	Culture duration and period (days)	Thawing success	Thawing passage
YCO-1	passage 28	394	success	passage 13
YCO-2	passage 41	708	success	passage 10
YCO-3	passage 36	564	success	passage 7
YCO-4	passage 28	490	success	passage 12
YCO-5	passage 34	440	success	passage 8
YCO-6	passage 36	464	success	passage 10
YCO-7	passage 25	447	success	passage 11
YCO-8	passage 42	459	success	passage 11
YCO-9	passage 36	496	success	passage 9
YCO-10	passage 31	460	success	passage 10
YCO-11	passage 26	438	success	passage 12
YCO-13	passage 18	248	success	passage 1
YCO-14	passage 14	168	success	passage 5
YCO-15	passage 36	801	success	passage 8
YCO-18	passage 13	205	success	passage 9
YCO-19	passage 15	251	success	passage 5

Supplementary Table 3. Previous intrahepatic cholangiocarcinoma organoid studies

	Our study	Markus H. Heim et al. Cell Reports, 2018[1]	Saito et al. Cell Reports, 2019[2]	Florin M. Selaru et al. JCI Insight, 2019[3]	Robert S. Warren et al. GI ASCO, 2020[4]
Sample type	Surgically resected and biopsy samples	Liver biopsy	Surgically resected samples	Surgically resected samples	Surgically resected samples
	Intrahepatic cholangiocarcinoma	Intrahepatic cholangiocarcinoma	Intrahepatic cholangiocarcinoma	Intrahepatic cholangiocarcinoma	Intrahepatic cholangiocarcinoma
Patient number	N =16	N = 3	N = 3<br>*Organoid line 1 was established using xenograft tissue derived from an ICC patient	N = 3	N = 4
Key topic	1. Verification of subgroup in intrahepatic cholangiocarcinoma using patient-derived organoid model<br>2. To find duct type-specific gene expression profile and targetable pathway as a therapeutic target	1. Organoids can be derived from tumor needle biopsies of liver cancers<br>2. Tumor organoids provide a tool for developing tailored therapies	1. Organoids as drug screening and research tools for the clarification of molecular pathogenesis and the discovery of biomarkers and therapeutic drugs	1. Drug screening to test intratumor and interpatient drug response heterogeneity	1. Organoid as drug screening tool and prediction of patient drug responses, high-throughput drug screening

1 Nuciforo, S. et al. Organoid Models of Human Liver Cancers Derived from Tumor Needle Biopsies. Cell Rep 24, 1363-1376 (2018).
2 Saito, Y. et al. Establishment of Patient-Derived Organoids and Drug Screening for Biliary Tract Carcinoma. Cell Rep 27, 1265-1276.e1264 (2019).
3 Li, L. et al. Human primary liver cancer organoids reveal intratumor and interpatient drug response heterogeneity. JCI Insight 4 (2019).
4 Antonia, R. J. et al. Patient-derived organoids for personalized drug screening in intrahepatic cholangiocarcinoma. Journal of Clinical Oncology 38, 581-581 (2020).

Supplementary Table 4. Conditioned media information (catalog number)

Reagent	Manufacturer	working conc.
B-27 SUPPLEMENT W/O VIT A (50X) 10 ML (cat no. 12587010)	Life Technologies	1X
N2 SUPPLEMENT (100X) 5ML (cat no. 17502048)	Life Technologies	1X
N-acetyl-L-cysteine (cat no. A7250)	Sigma-Aldrich	1mM
R-spondin conditioned medium		10%
Nicotinamide (cat no. N0636)	Sigma-Aldrich	10mM
[Leu15]-gastrin I human (cat no. G9145)	Sigma-Aldrich	10nM
Recombinant human EGF (cat no. AF-100-15)	Peprotech	50ng/ml
Recombinant human FGF10 (cat no. 100-26)	Peprotech	100ng/ml
rhHGF (cat no. 100-39)	Peprotech	25ng/ml
Forskolin (cat no. 1099)	Tocris	10uM
A83-01 (TGFβ inhibitor) (cat no. 2939)	Tocris	5uM
primocin (cat no. ant-pm-1)	invivoGen	50ug/ml
ADVANCED D-MEM/F-12 500ML (cat no. 12634010)	Life Technologies	

Supplementary Table 5. Immunohistochemistry antibody information

Antibody	Product name	Manufacturer	Clone	Dilution
S100P	S100P polyclonal antibody (cat. no. PA5-80992)	Thermo Fisher scientific, MA, USA		1:1000
CD56	CD56 monoclonal antibody (cat. no. 07-5603)	Thermo Fisher scientific, MA, USA	123C3	1:200
N-cadherin (CDH2)	CDH2 mouse monoclonal antibody (cat.no. UM500023)	OriGene Technologies, Inc., MD, USA	UMAB23	1:200
p53	p53 mouse monoclonal antibody (cat.no. sc-126)	Santa Cruz Biotechnology, Inc., Texas, USA	DO-1	1:200

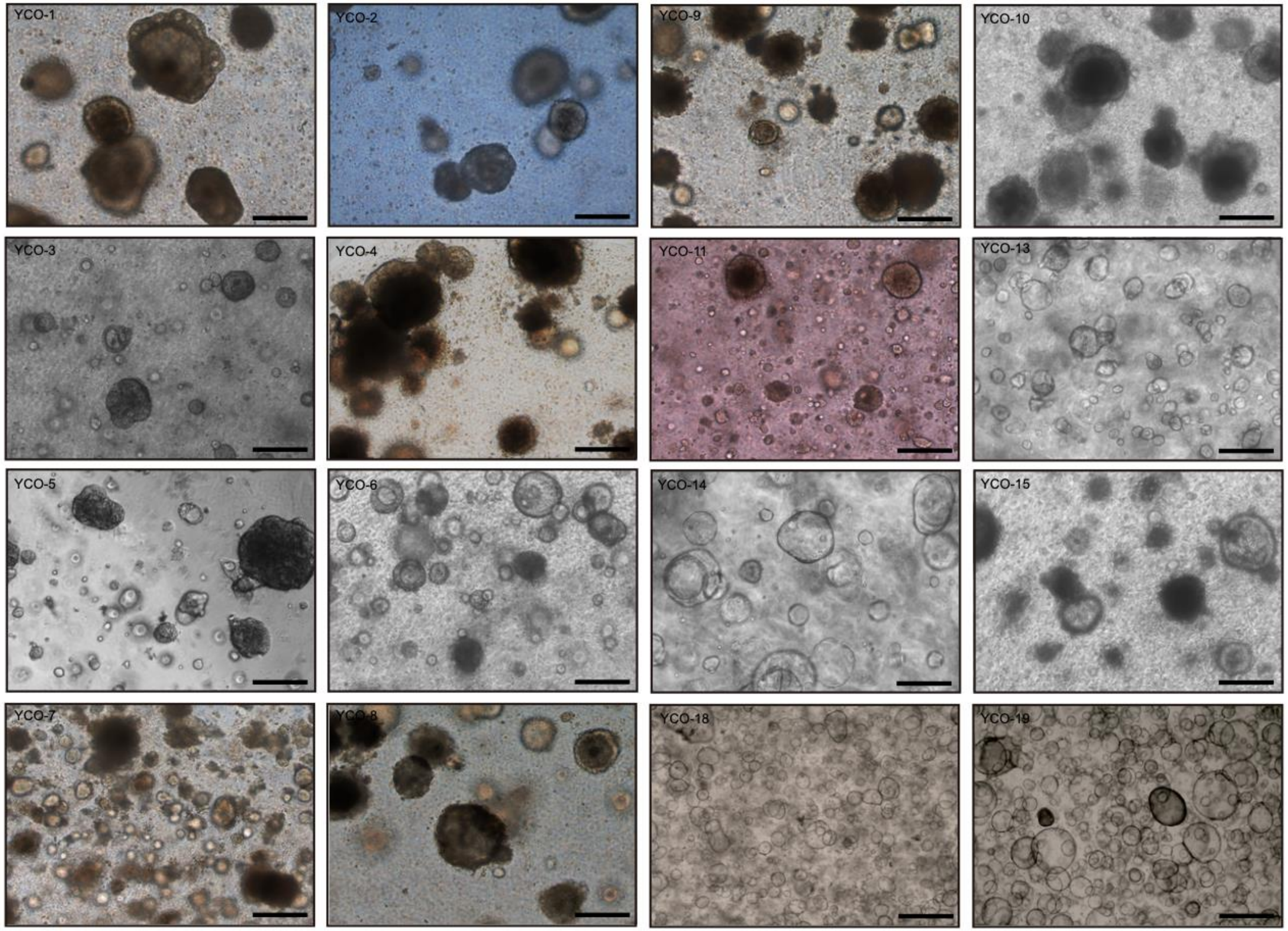

Supplementary figure 1. ICC organoids in bright field images. Organoids has various morphology such as cystic thick wall, compact type, or mixes type (*n*=16). scale bar, 200 μm.

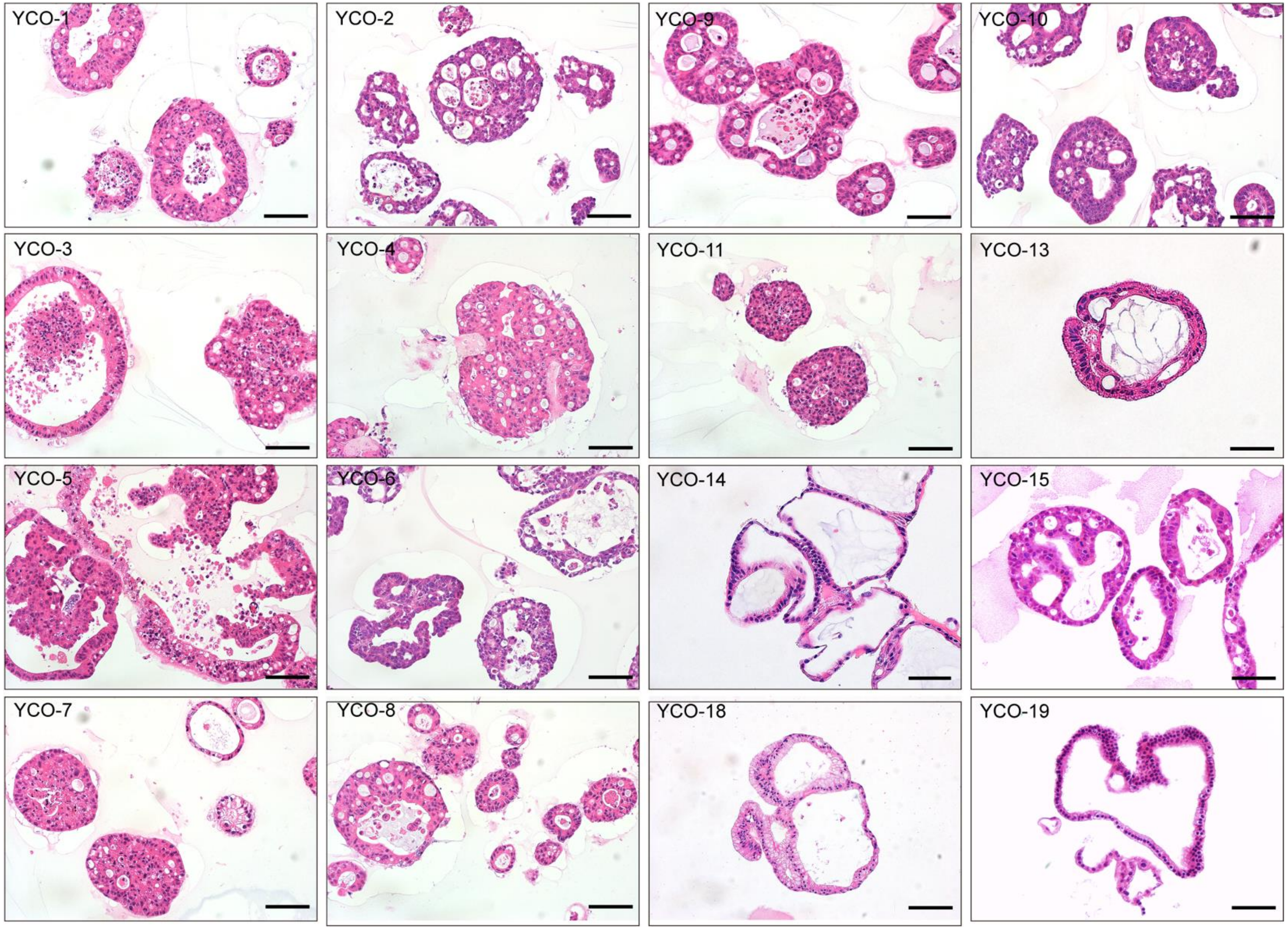

Supplementary figure 2. H&E stain of all organoid samples. The organoids were compact shaped or irregularly shaped with thickened cyst-like structures on H&E images (n=16). Scale bar, 100 µm.

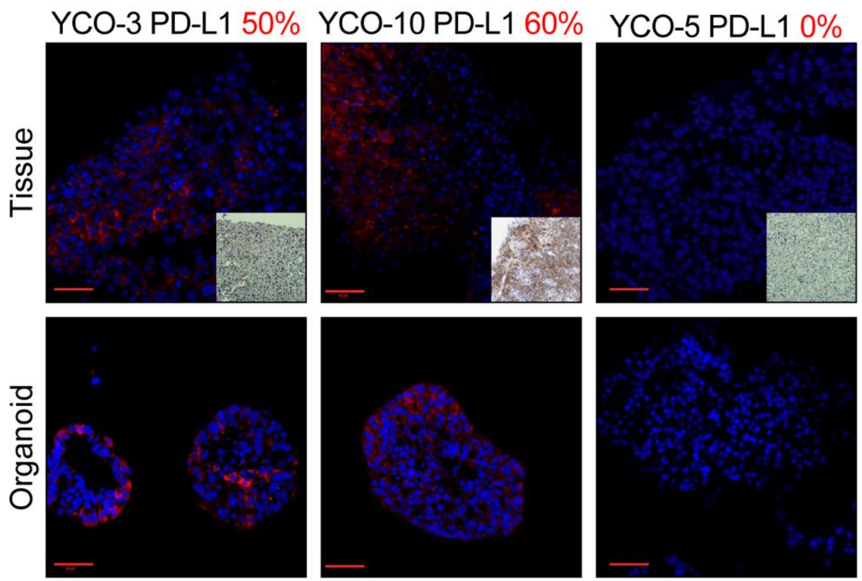

Supplementary figure 3. Similarity of immunologic marker PD-L1 expression in organoids developed from tumor epithelial cell. The expression of PD-L1 level in primary tumor tissue was matched with it of tumor organoids. In patient YCO-3, high PD-L1 expression (50%) was noted in both tissue and organoids; by comparison PD-L1 expression was undetectable (0%) in patient YCO-5 (n=3). Scale bar, 100 µm.

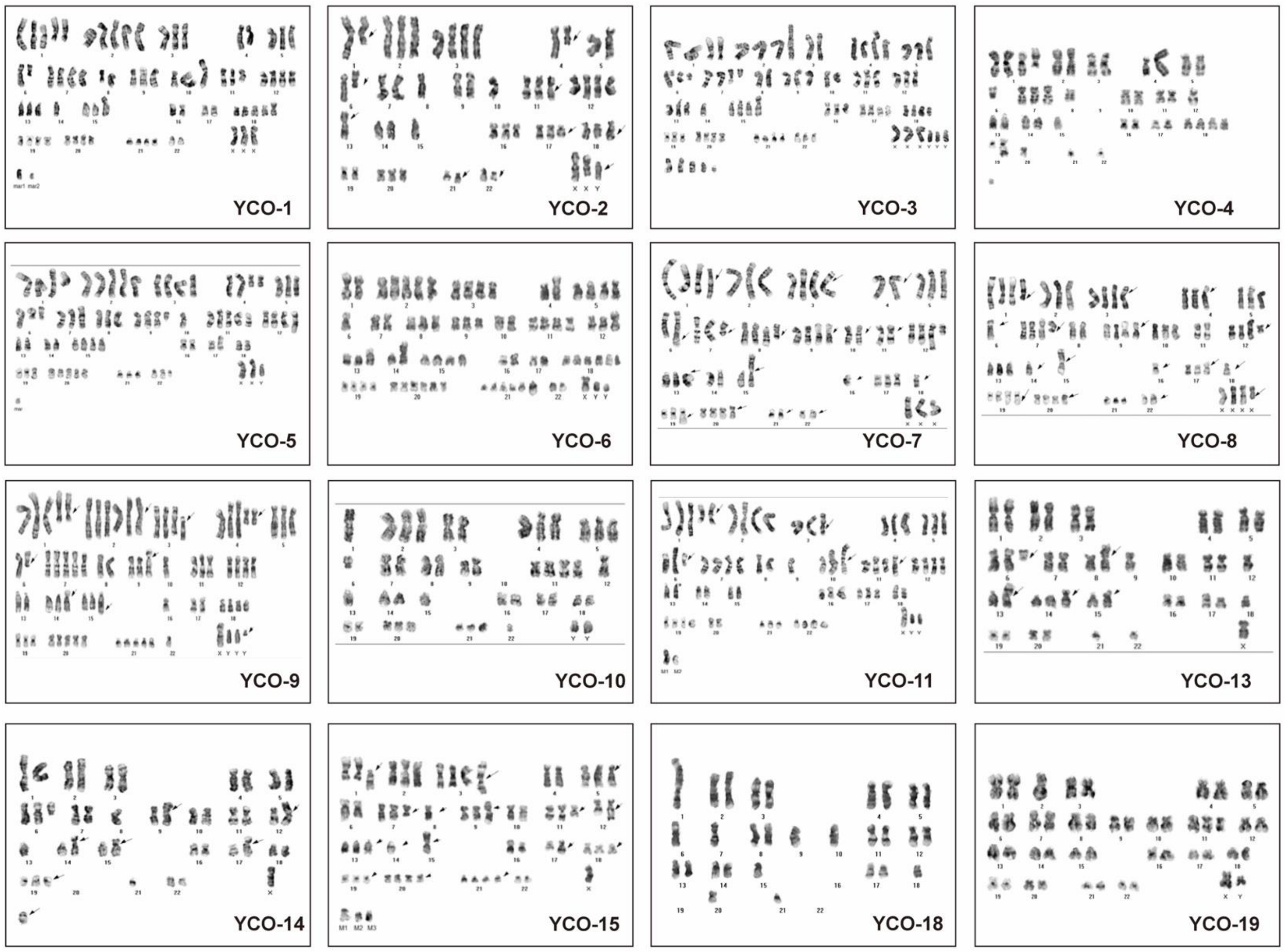

Supplementary figure 4. Karyotype of organoids. The organoids showed aneuploidy in karyotyping (*n*=16).

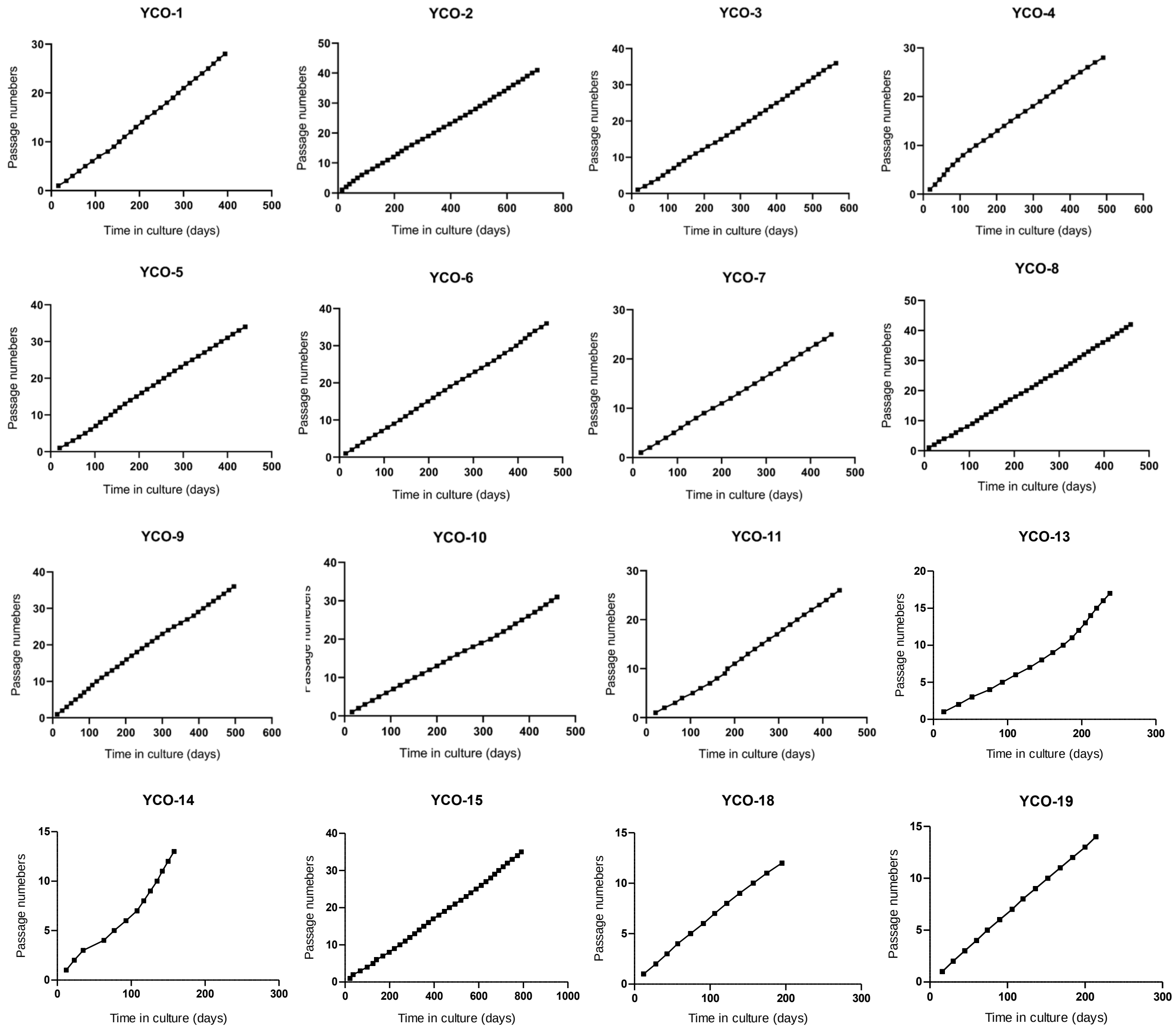

Supplementary figure 5. Cancer organoids growth curve and kinetics. Compared to control normal organoids, cancer organoids grew continuously without senescence. The last culture passages ranged between 13 and 42. The median culture duration and period were about 453 days (168-708 days). Source data are provided as a Source Data file.

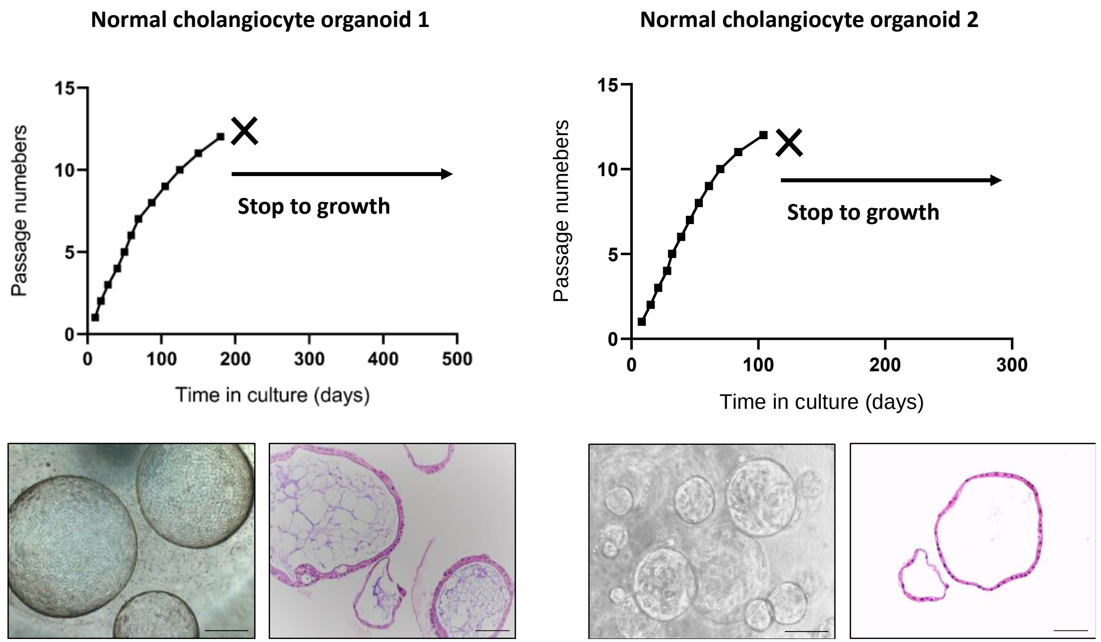

Supplementary figure 6. Supplementary figure 6. Normal biliary organoids growth curve and kinetics. Non-CCA bile duct organoids have a homogeneous, cyst-like hollow structures, and they grow slowly after ten passages (*n*=2). Scale bar, 100 µm. Source data are provided as a Source Data file.

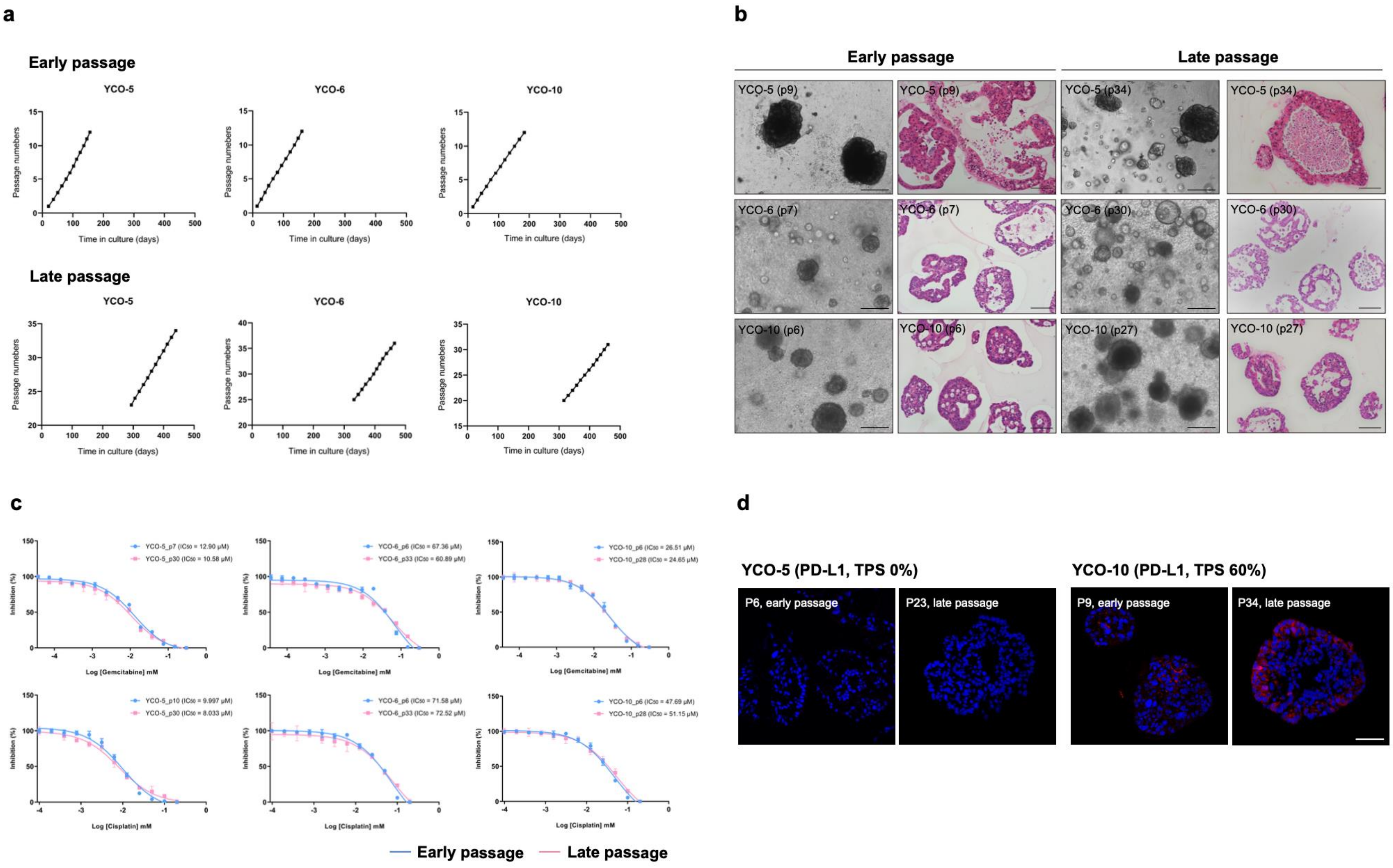
a
Early passage
YCO-5
YCO-6
YCO-10
Passage numebers
Time in culture (days)
Late passage
b
Early passage
Late passage
YCO-5 (p9)
YCO-5 (p34)
YCO-6 (p7)
YCO-6 (p30)
YCO-10 (p6)
YCO-10 (p27)
c
YCO-5_p7 (IC50 = 12.90 μM)
YCO-5_p30 (IC50 = 10.58 μM)
YCO-6_p6 (IC50 = 67.36 μM)
YCO-6_p33 (IC50 = 60.89 μM)
YCO-10_p6 (IC50 = 26.51 μM)
YCO-10_p28 (IC50 = 24.65 μM)
Inhibition (%)
Log [Gemcitabine] mM
YCO-5_p10 (IC50 = 9.997 μM)
YCO-5_p30 (IC50 = 8.033 μM)
YCO-6_p6 (IC50 = 71.58 μM)
YCO-6_p33 (IC50 = 72.52 μM)
YCO-10_p6 (IC50 = 47.69 μM)
YCO-10_p28 (IC50 = 51.15 μM)
Log [Cisplatin] mM
Early passage
Late passage
d
YCO-5 (PD-L1, TPS 0%)
YCO-10 (PD-L1, TPS 60%)
P6, early passage
P23, late passage
P9, early passage
P34, late passage

Supplementary figure 7. Comparison between early and late passages of organoids. We were able to culture and stably maintain the organoids derived from cancer tissues stably, and found no significant difference in the morphological changes and histology, growth rate, immunology (PD-L1 expression on organoids), and response to drug by the organoids between early and late passages. a The growth rate was similar between early and late cultured organoids. b The phenotype and histology were still similar between early passage and late passage (*n*=6, 3 samples in early and late passage, respectively). Scale bar, 100 µm. c YCO-5 was sensitive to gemcitabine (IC50=12.9uM). Late ICC organoid of YCO-5 was still sensitive to gemcitabine (IC50=10.58uM). (*n*=6, 3 samples in early and late passage, respectively). Data are presented as mean ± SD. d The expression level of immunologic marker PD-L1 was well-sustained (*n*=4, 2 samples in early and late passage, respectively). Scale bar, 100 µm. Source data are provided as a Source Data file.

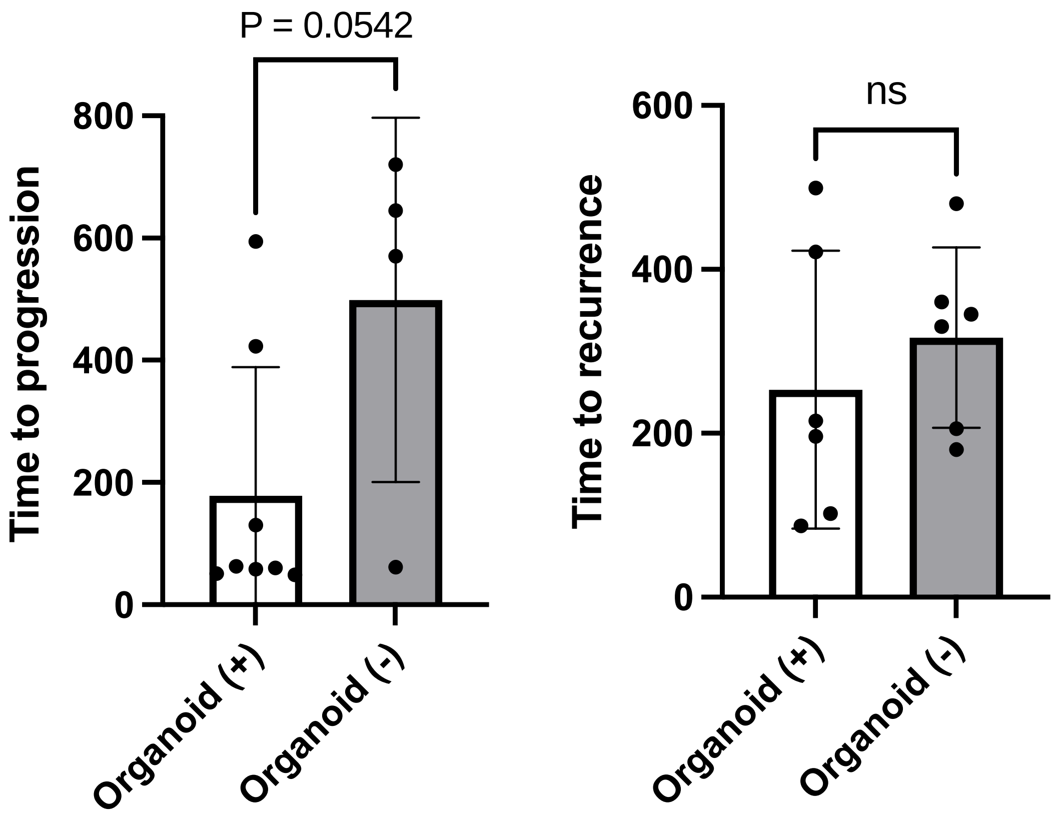

Supplementary figure 8. The organoids establishment rate associated with the aggressive features of the tumor. Patients with established organoids showed shorter time to progression (178.5 ± 210.3 days vs. 499.1 ± 298.1 days, *P* value = 0.054) or recur free survival (253.3 ± 169.6 days vs. 316.8 ± 110.1 days, *P* value = 0.460) than those who failed to establish organoids, but statistically not significant. Time to progression, n = 8 and 4 biologically independent samples for each group (*n*=12). Time to recurrence, n = 6 biologically independent samples for each group (*n*=12). Data are presented as mean ± SD. Statistical analysis was performed using 2-tailed Students' t-test. ns., not significant. Source data are provided as a Source Data file.

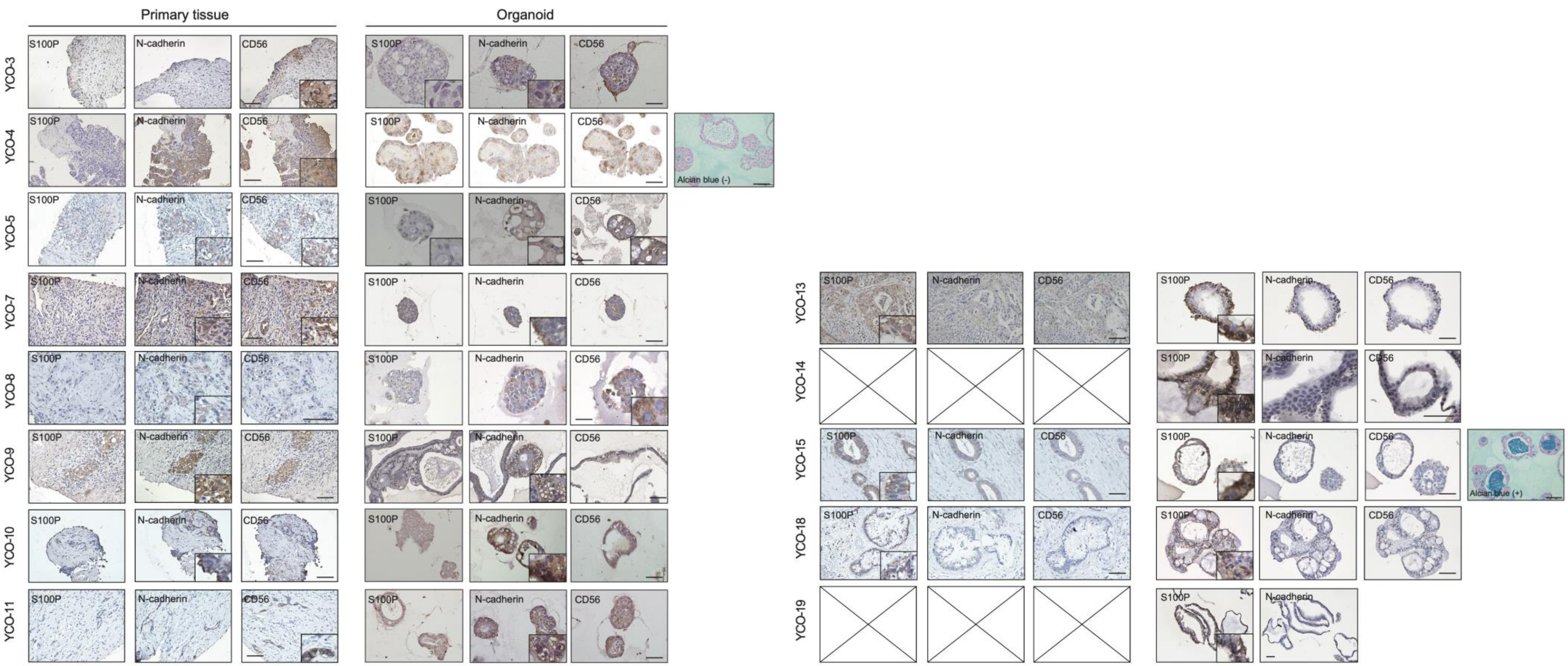

Supplementary figure 9. IHC stain for three markers (S100P, N-cadherin, and CD56) on the tissue and organoids slides. Intrahepatic cholangiocarcinoma organoids can be divided into large duct type (S100P +) and small duct type (N-cadherin +, CD56 +) (*n*=16). In organoids, YCO-4 showed S100P (+) and CD 56 (+), therefore we also stained by Alcian blue (positive in large duct type) which was negative in YCO-4 (*n*=2). Scale bar, 100 μm.
