# Supplementary material for: Integrative analysis of multiple genomic data from intrahepatic cholangiocarcinoma organoids enables tumor subtyping
Source: Nat Commun. 2023 Jan 16;14:237. doi: 10.1038/s41467-023-35896-4 (PMC9842736; doi:10.1038/s41467-023-35896-4)
Supplement: Supplementary file 2 — Reporting Summary [file 41467_2023_35896_MOESM2_ESM.pdf]

nature portfolio

Corresponding author(s): Jun Yong Park, NCOMMS-22-08035

Last updated by author(s): Dec 4, 2022

Reporting Summary

Nature Portfolio wishes to improve the reproducibility of the work that we publish. This form provides structure for consistency and transparency in reporting. For further information on Nature Portfolio policies, see our Editorial Policies and the Editorial Policy Checklist.

Statistics

For all statistical analyses, confirm that the following items are present in the figure legend, table legend, main text, or Methods section.

n/a	Confirmed	
☐	☒	The exact sample size (n) for each experimental group/condition, given as a discrete number and unit of measurement
☐	☒	A statement on whether measurements were taken from distinct samples or whether the same sample was measured repeatedly
☐	☒	The statistical test(s) used AND whether they are one- or two-sided<br>*Only common tests should be described solely by name; describe more complex techniques in the Methods section.*
☒	☐	A description of all covariates tested
☒	☐	A description of any assumptions or corrections, such as tests of normality and adjustment for multiple comparisons
☐	☒	A full description of the statistical parameters including central tendency (e.g. means) or other basic estimates (e.g. regression coefficient) AND variation (e.g. standard deviation) or associated estimates of uncertainty (e.g. confidence intervals)
☒	☐	For null hypothesis testing, the test statistic (e.g. F, t, r) with confidence intervals, effect sizes, degrees of freedom and P value noted<br>*Give P values as exact values whenever suitable.*
☒	☐	For Bayesian analysis, information on the choice of priors and Markov chain Monte Carlo settings
☒	☐	For hierarchical and complex designs, identification of the appropriate level for tests and full reporting of outcomes
☒	☐	Estimates of effect sizes (e.g. Cohen's d, Pearson's r), indicating how they were calculated

Our web collection on statistics for biologists contains articles on many of the points above.

Software and code

Policy information about availability of computer code

Data collection	For IHC, sections were counterstained with hematoxylin and imaged under a BX51 microscope (Olympus, Tokyo, Japan).<br>Slide section: Leica HistoCore BIOCUT (Leica Biosystems, USA)<br>Fluorescent images: Zeiss LSM 780 (Zeiss, Germany)<br>Transcriptomic data and DNA-seq data were performed using NovaSeq6000 (Illumina, USA)

Data analysis	DNA-seq Image analysis was performed using NovaSeq6000 control Software version 1.3.1, and output base-calling data were de-multiplexed with bcl2fastq version v2.20.0.422, generating fastQC files. Sequencing reads were first mapped/aligned to reference genome hg19 using the Burrows-Wheeler Aligner (BWA). Pileup and variant calling from the aligned sequence reads were performed using BCFtools 1.12, and called variants were annotated using Ensembl Variant Effect Predictor (VEP) release 105. The resulting annotated variants were filtered according to the following criteria: depth (DP) > 50; alternate allele count (AC) > 5; and allele frequency (AF) > 0.05. The filtered variants and their effects on amino acid sequence were visualized using Maftools 2.10.0. RNA sequencing was performed using a TruSeq Stranded mRNA Sample Prep Kit (Illumina). Adapter sequences and ends of reads with a Phred quality score less than 20 were trimmed, and reads shorter than 50 bp were simultaneously removed using cutadapt v.2.8. Filtered reads were mapped to the species reference genome using the aligner, STAR v.2.7.1a, following ENCODE standard options, with application of the "quantMode TranscriptomeSAM" option for estimation of transcriptome expression level. Gene expression levels were estimated using RSEM v.1.3.1. For Transcriptomic analysis, gene sets from the Molecular Signatures Database (MSigDB) were used for gene set enrichment analysis (GSEA), and protein-protein interactions for transcription factors were analyzed using the web-based software, Enrichr (web-based software) R packages (ver 3.6.2). Differentially expressed genes (DEGs) were analyzed using the DESeq2 algorithm. Differentially expressed genes (DEGs) were analyzed using the DESeq2 algorithm and were defined based on a P-value < 0.05 and log2 fold change > 1. For the data analysis and visualization, GraphPad Prism (Version 9.1.0), Adobe Illustrator CC 2022, R (version 3.6.2) packages were used.

For manuscripts utilizing custom algorithms or software that are central to the research but not yet described in published literature, software must be made available to editors and reviewers. We strongly encourage code deposition in a community repository (e.g. GitHub). See the Nature Portfolio guidelines for submitting code & software for further information.

Data

Policy information about availability of data

All manuscripts must include a data availability statement. This statement should provide the following information, where applicable:

- Accession codes, unique identifiers, or web links for publicly available datasets
- A description of any restrictions on data availability
- For clinical datasets or third party data, please ensure that the statement adheres to our policy

The transcriptome data used in this study are available in the GEO database under accession code GSE215997(https://www.ncbi.nlm.nih.gov/geo/query/acc.cgi?acc=GSE215997). The whole exome sequencing data used in this study are available in the GEO database under accession code GSE220940 (https://www.ncbi.nlm.nih.gov/geo/query/acc.cgi?acc=GSE220940). Gene sets can be downloaded from MSigDB (https://www.gsea-msigdb.org/gsea/msigdb/). The remaining data are available within the Article, Supplementary Information or Source data file. Source data are provided with this paper.

Human research participants

Policy information about studies involving human research participants and Sex and Gender in Research.

Reporting on sex and gender	Of the 16 patients, 11 (68.8%) were male and 5 (31.2%) were female.
Population characteristics	All patients were diagnosed with cholangiocarcinoma, as confirmed by histological analysis. Median age was 64 years old. Cancer tissues, matching normal tissues, and blood samples were collected from those patients
Recruitment	All patients have been diagnosed with cholangiocarcinoma and treated according to routine practice. The participants were recruited after acquiring their informed consents. No bias on selecting patients existed. To see patients characteristics, please see Table 1.
Ethics oversight	The collection and use of human samples were approved by the Ethics Committee of Severance hospital of the Yonsei University of college of medicine, following the Declaration of Helsinki ethical guidelines. All patients provided informed consent, and procedures were approved by the Institutional Review Board according to ethical guidelines.

Note that full information on the approval of the study protocol must also be provided in the manuscript.

Field-specific reporting

Please select the one below that is the best fit for your research. If you are not sure, read the appropriate sections before making your selection.

☒ Life sciences ☐ Behavioural & social sciences ☐ Ecological, evolutionary & environmental sciences

For a reference copy of the document with all sections, see nature.com/documents/nr-reporting-summary-flat.pdf

Life sciences study design

All studies must disclose on these points even when the disclosure is negative.

Sample size	All patients with intrahepatic cholangiocarcinoma that have been treated at the Severance hospital were included in the present study. No statistical methods were used to predetermine sample size. Sample size was chosen based on the established intrahepatic cholangiocarcinoma organoids.

Data exclusions	No data or animals were excluded from analysis.
Replication	All experiments were repeated at least three times showing similar results. All attempts at replication were successful and number is described in legends. IHC and IF were performed independently in each samples.
Randomization	Patients were randomly recruited in this study, and enrolled according to their ICC organoid type.
Blinding	Investigators were blinded during all histological analyses, mouse experiments, and drug sensitivity test. Blinding was performed without information of the type of ICC organoids.

Reporting for specific materials, systems and methods

We require information from authors about some types of materials, experimental systems and methods used in many studies. Here, indicate whether each material, system or method listed is relevant to your study. If you are not sure if a list item applies to your research, read the appropriate section before selecting a response.

Materials & experimental systems

n/a	Involved in the study
☐	☒ Antibodies
☒	☐ Eukaryotic cell lines
☒	☐ Palaeontology and archaeology
☐	☒ Animals and other organisms
☒	☐ Clinical data
☒	☐ Dual use research of concern

Methods

n/a	Involved in the study
☒	☐ ChIP-seq
☒	☐ Flow cytometry
☒	☐ MRI-based neuroimaging

Antibodies

Antibodies used	S100P polyclonal antibody (invitrogen, cat. no. PA5-80992, lot no. WA3157015), CD56 monoclonal antibody (invitrogen, cat. no. 07-5603, clone name 123C3, lot no. VB301788), N-cadherin (CDH2) mouse monoclonal antibody (origene, cat.no. UM500023, clone name UMAB23, lot no. A001), AFP monoclonal antibody (Abcam, cat. no. ab3980, clone name AFP-01, lot no. GR3284459-3, 1:200), KRT19 monoclonal antibody (Abcam, cat. no. ab133496, clone name EPNCIR127B, lot no. GR3318533-1, 1:1000), KRT7 monoclonal antibody (Abcam, cat. no. ab181598, clone name EPR17078, lot no. GR3214132-6, 1:500), albumin polyclonal antibody (Bethyl Laboratories, cat. no. A80-129A, lot no. 0000057593, 1:200), vimentin monoclonal antibody (Cell Signaling, cat. no. 5741S, clone name D21H3, lot no. 45, 1:400), SOX9 monoclonal antibody (Abcam, cat. no. ab185966, clone name EPR14335-78, lot no. GR3241181-4, 1:1000), and PD-L1 monoclonal antibody (Cell Signaling, cat. no. 13684S, clone name E1L3N, lot no. 13, 1:1000).<br>Alexa Fluoro647 donkey anti-rabbit IgG (Invitrogen, cat. no. A31573, lot no. 2181018, 1:2000), Alexa Fluoro488 donkey anti-mouse IgG (Invitrogen, cat. no. A21202, lot no. 2147618, 1:2000), Alexa Fluoro594 donkey anti-goat IgG (Invitrogen, cat. no. A11058, lot no. 1608643, 1:2000)
Validation	All purchased antibodies were used according to the manufacturer's instructions. All antibodies used are commercially available and validated by the manufacturers. The validation information for the species and application are following;<br>S100P (human & Immunohistochemistry (Paraffin), Immunocytochemistry (ICC/IF)), CD56 (Virus, Human, Mouse & Western Blot, Immunohistochemistry, Flow Cytometry, Immunoprecipitation), N-cadherin (Human, Mouse, Rat & Flow Cytometry, Immunofluorescence, Immunohistochemistry, Western Blot), AFP (Human & Western Blot, Sandwich ELISA, ELISA, Immunocytochemistry (ICC/IF), Immunoprecipitation), KRT19 (Mouse & Western Blot, Immunohistochemistry (Paraffin)), KRT7 (Mouse, Rat, Human & Western Blot, Immunohistochemistry, Immunocytochemistry, Flow Cytometry),<br>albumin (Human & Western Blot, ELISA, Immunohistochemistry, Immunocytochemistry), vimentin (Mouse, Rat, Human & Western Blot, Immunohistochemistry, Immunocytochemistry, Flow Cytometry), SOX9 (Human, Mouse, Rabbit, Monkey & Western Blot, Immunoprecipitation, Immunohistochemistry, Immunocytochemistry, Flow Cytometry), PD-L1 (Human & Western Blot, Immunoprecipitation, Immunohistochemistry, Flow Cytometry), Alexa Fluoro647 donkey anti-rabbit IgG (Rabbit & Western Blot, Immunohistochemistry, Immunocytochemistry), Alexa Fluoro488 donkey anti-mouse IgG (Mouse & Immunohistochemistry, Immunocytochemistry), Alexa Fluoro594 donkey anti-goat IgG (Goat & Immunohistochemistry, Immunocytochemistry, Flow Cytometry)

Animals and other research organisms

Policy information about studies involving animals; ARRIVE guidelines recommended for reporting animal research, and Sex and Gender in Research

Laboratory animals	5-week-old male NOD/SCID (severe combined immunodeficiency) mice (Charles River Laboratories, Tokyo, Japan).<br>All mice were housed with following housing condition. Dark/light cycle: 12hr /12hr (8am ~ 8pm light on, 8pm ~ 8am light off) temperate: 22 degrees Celsius (± 2), humidity: 50% (± 10%)
Wild animals	This study did not involve wild animals.

Reporting on sex	Only male animals were used (male NOD/SCID mice from Charles River Laboratories, Tokyo, Japan).
Field-collected samples	This study did not involve samples collected in the field
Ethics oversight	Animals were housed at the Yonsei University animal care facility according to institutional guidelines. All experiments were performed in accordance with approved animal use procedures.

Note that full information on the approval of the study protocol must also be provided in the manuscript.
